# Kidney Function Decline After COVID-19 Infection

**DOI:** 10.1001/jamanetworkopen.2024.50014

**Published:** 2024-12-26

**Authors:** Viyaasan Mahalingasivam, Anne-Laure Faucon, Arvid Sjölander, Alessandro Bosi, Ailema González-Ortiz, Stefania Lando, Edouard L. Fu, Dorothea Nitsch, Annette Bruchfeld, Marie Evans, Kevin Wing, Kathryn E. Mansfield, Laurie Tomlinson, Juan-Jesús Carrero

**Affiliations:** 1 Department of Non-Communicable Disease Epidemiology, London School of Hygiene and Tropical Medicine, London, United Kingdom; 2 Department of Medical Epidemiology and Biostatistics, Karolinska Institutet, Stockholm, Sweden; 3 Department of Nephrology and Transplantation, Barts Health National Health Service Trust, London, United Kingdom; 4 Department of Clinical Epidemiology, Institut National de la Santé et de la Recherche Médicale U1018, Paris-Saclay University, Villejuif, France; 5 Translational Research Center, Instituto Nacional de Pediatria, Mexico City, Mexico; 6 Department of Clinical Epidemiology, Leiden University Medical Center, Leiden, the Netherlands; 7 UK Kidney Association, Bristol, United Kingdom; 8 Department of Health, Medicine and Caring Science, Linköping University, Linköping, Sweden; 9 Unit of Renal Medicine, Department of Clinical Science, Intervention and Technology, Karolinska Institutet, Stockholm, Sweden; 10 Unit of Renal Medicine, Karolinska University Hospital Huddinge, Stockholm, Sweden; 11 Public Health, School of Health and Wellbeing, University of Glasgow, Glasgow, United Kingdom

## Abstract

**Importance:**

COVID-19 infection has been associated with acute kidney injury. However, its possible association with long-term kidney function is not well understood.

**Objective:**

To investigate whether kidney function decline accelerated after COVID-19 compared with after other respiratory tract infections.

**Design, Setting, and Participants:**

This cohort study used linked data from the Stockholm Creatinine Measurements (SCREAM) Project between February 1, 2018, and January 1, 2022, in Stockholm, Sweden. All hospitalized and nonhospitalized adults in the database with at least 1 estimated glomerular filtration rate (eGFR) measurement in the 2 years prior to a COVID-19 positive test result or pneumonia diagnosis were selected. Statistical analyses were conducted between June 2023 and October 2024.

**Exposure:**

COVID-19 and pneumonia (including influenza).

**Main Outcomes and Measures:**

Mean annual change in eGFR after COVID-19 and after pneumonia was calculated with a linear regression model.

**Results:**

The COVID-19 cohort comprised 134 565 individuals (74 819 females [55.6%]; median [IQR] age, 51 [37-64] years). The pneumonia cohort consisted of 35 987 individuals (19 359 females [53.8%]; median [IQR] age, 71 [56-81] years). The median (IQR) baseline eGFR was 94 (79-107) mL/min/1.73m^2^ for the COVID-19 cohort and 79 (61-92) mL/min/1.73m^2^ for the pneumonia cohort. After adjustment for covariates, both infections demonstrated accelerated annual eGFR decline, with greater magnitude of decline after COVID-19 (3.4% [95% CI, 3.2%-3.5%] after COVID-19; 2.3% [95% CI, 2.1%-2.5%] after pneumonia). This decline was more severe among individuals hospitalized for COVID-19 (5.4%; 95% CI, 5.2%-5.6%) but remained similar among those hospitalized for pneumonia.

**Conclusions and Relevance:**

This cohort study found an association between COVID-19 and accelerated decline in kidney function, particularly after hospitalization, compared with pneumonia. People who were hospitalized for COVID-19 should receive closer monitoring of kidney function to ensure early diagnosis and optimized management of chronic kidney disease to effectively prevent complications and further decline.

## Key Points

**Question:**

Is there an acceleration in kidney function decline after COVID-19 infection and does it differ from after other lung infections?

**Findings:**

In this cohort study of 134 565 individuals with COVID-19 and 35 987 individuals with pneumonia, there was a greater annual decline in kidney function after COVID-19 compared with after pneumonia. This decline was greater among people who were hospitalized for COVID-19.

**Meaning:**

Findings of this study suggest an association between COVID-19 and accelerated kidney function decline, especially among people who were hospitalized; these people need closer monitoring of kidney function for early diagnosis and management of chronic kidney disease.

## Teaser

This cohort study examines the annual decrease in estimated glomerular filtration rates among adults diagnosed with COVID-19 and pneumonia in Stockholm, Sweden.

## Introduction

SARS-CoV-2 (the virus that causes COVID-19) may directly or indirectly affect the kidney,^1^ with acute kidney injury (AKI) observed in approximately 30% of COVID-19 hospitalizations.^2,3,4,5^ While histopathological series have raised the possibility of irreversible damage,^1,6^ epidemiological inquiry into long-term outcomes has been limited. Existing studies have generally found increased adverse kidney outcomes after COVID-19 compared with individuals without the infection, with conflicting findings among individuals with influenza.^7,8,9,10^ Besides the differences in settings, definitions, and duration of follow-up, a common limitation of prior studies has been the lack of consideration of preexisting kidney function trajectory, which may explain both postinfection decline and the need for function monitoring.

To address this missing component, we conducted this study using routinely collected data from the Stockholm region of Sweden. Our objective was to investigate whether kidney function decline compared with preexisting estimated glomerular filtration rate (eGFR) trajectories accelerated after COVID-19 infection. Because severe COVID-19 usually presents as a viral pneumonia, we also investigated whether the acceleration in kidney function decline differed from after pneumonia caused by other pathogens; similar to severe COVID-19, preexisting kidney disease is a risk factor for pneumonia.^11^

## Methods

### Data Source

For this cohort study, we used linked health record data from the Stockholm Creatinine Measurements (SCREAM) Project (eAppendix 1 in Supplement 1).^12^ The Regional Ethical Review Board in Stockholm approved the study and deemed the informed consent requirement unnecessary because the data used were deidentified at the Swedish Board of Health and Welfare. This report follows the Strengthening the Reporting of Observational Studies in Epidemiology (STROBE)^13^ and the Reporting of Studies Conducted Using Observational Routinely Collected Data (RECORD)^14^ reporting guidelines.

### Study Population

#### COVID-19 Cohort

We included all adults (aged ≥18 years) with their first recorded positive result from a COVID-19 polymerase chain reaction or antigen test from February 1, 2020, to January 1, 2022, as recorded weekly by the Swedish Public Health Agency. During this period, the dominant COVID-19 variants in Sweden were the wild-type, Alpha, and Delta; the Omicron (BA.1) variant became dominant only at the end of December 2021. We classified individuals as hospitalized if a hospitalization episode occurred within 28 days of their first recorded positive result or if their condition was coded for COVID-19 using *International Statistical Classification of Diseases and Related Health Problems, Tenth Revision* (*ICD-10*) code U07 (eAppendix 2 in Supplement 1) during a hospitalization episode.

#### Prepandemic Pneumonia Cohort

We selected adults with a diagnosis of pneumonia (including *ICD-10* coded influenza) between February 1, 2018, and January 1, 2020 (eAppendix 2 in Supplement 1). We chose pneumonia as a comparator given that pneumonia is the predominant indication for COVID-19 hospitalization, and we chose a period prior to the pandemic to avoid misclassification.

The index date was the date of COVID-19 or pneumonia diagnosis. Together, these cohorts formed the primary study dataset. Cohorts were not mutually exclusive; that is, individuals who had prepandemic pneumonia were eligible for inclusion in the COVID-19 cohort. If an individual had more than 1 episode of either COVID-19 or pneumonia during the follow-up period, we considered only the first episode. The exclusion criterion was receipt of dialysis at index date or not having any serum creatinine measurements within 2 years before the index date. We conducted this analysis between June 2023 and October 2024.

### Outcomes

The primary study outcome was the mean annual change in eGFR slopes before vs after each infection. The secondary study outcome was the annual change in postinfection eGFR slopes after COVID-19 or pneumonia, accounting for differences in confounders. eGFR was calculated using the 2009 CKD-EPI (Chronic Kidney Disease Epidemiology Collaboration) equation without adjustment for ethnicity.^10,11^ Preinfection eGFR slopes were constructed from all outpatient measurements up to 2 years prior to the index date to be the balance between kidney function decline and no substantial change due to periods of stable eGFR over a longer time frame. We purposely excluded measurements performed within 1 month before and 2 months after the index date from the computation of slopes on the assumption that these measurements could have been affected by acute illness (eFigure 1 in Supplement 1).

The tertiary study outcome was time to 25% reduction in eGFR, including incident kidney replacement therapy (ie, dialysis or kidney transplant). Kidney replacement therapy date was ascertained through the Swedish Renal Registry linkage. To reduce outcome misclassification owing to intrinsic variability in eGFR and to confirm that eGFR declines were sustained, we used a linear interpolation method.^15,16^ For each individual, we fitted a linear regression through all outpatient measurements and interpolated the outcome when the regressed eGFR had declined by 25% from the initial measurement.

### Covariates

We established the status of covariates at index date. Covariates included demographic, socioeconomic, and clinical factors. Socioeconomic variables were obtained from the Longitudinal Integrated Database for Health Insurance and Labour Market Studies^17^ and included highest educational level (categorized as compulsory school [up to age 16 years], upper secondary school [up to age 19 years], university, or missing data) and annual income (categorized into lowest, middle, and highest tertiles). To ascertain eGFR at index date (baseline kidney function), we used the mean of all eGFR records between 1 and 18 months prior to the index date. We identified history of comorbid conditions before the index date using *ICD-10* codes (eAppendix 2 in Supplement 1). Renin-angiotensin system inhibitor (RASi) use was ascertained via pharmacy fills captured through the National Prescribed Drug Register linkage.^18^

The recording of ethnicity is not permitted in Sweden. Data on body mass index was also not available.

### Statistical Analysis

We reported descriptive statistics as medians and IQRs for continuous variables and as counts and percentages for categorical variables. We also described the frequency of creatinine tests before and after COVID-19 and pneumonia.

To estimate the mean annual change in eGFR before vs after each infection (primary study outcome), we used a linear regression model.^15^ In this model, we regressed each eGFR measurement on the following variables: time (date of measurement), period (before or after infection), and infection (pneumonia or COVID-19) (eAppendix 3 in Supplement 1). We fitted the model with 2 covariates: age and sex; other covariates were extracted at index date and therefore could not be included. We used this model to estimate the mean annual change in eGFR for all 4 combinations of period and infection as well as the difference in the mean annual change in eGFR between the period before and after infection.

To estimate the mean annual change in eGFR after pneumonia and COVID-19 and the difference between these, we fitted another linear regression adjusted for age, sex, annual income, educational level, diabetes, hypertension, cardiovascular diseases, nonhematological cancer, immunosuppression, history of AKI, previous pneumonia, number of hospital admissions in the preceding 5 years, RASi use in the preceding 6 months, eGFR slope before infection, baseline eGFR, and number of creatinine tests used in the computation of preinfection eGFR slopes. These covariates were selected based on a directed acyclic graph (eFigure 2 in Supplement 1). Per definition, this analysis can be performed only in the subset of participants from the primary dataset for whom eGFR measurements after infection were available (the secondary dataset). This analysis is equivalent to a difference-in-difference (DID) approach; specifically, the eGFR slopes after COVID-19 and pneumonia respectively are differences over time, and the differences between these slopes can then be interpreted as a DID (ie, free from all time-stationary and time-varying confounders). As in a usual DID analysis, this interpretation hinges on the assumption that the implications of both time-stationary and time-varying confounders is the same for both groups. In both models, we used a clustered sandwich estimator of variance to account for the correlation of repeated eGFR measurements from the same individual.^19^ While a linear mixed-effects model is more efficient, it is less robust than a clustered sandwich estimator since it makes more modeling assumptions (eg, it models the correlation structure for repeated measurements). Because we had a large dataset, we aimed for robustness rather than efficiency. We calculated the annual percentage decline in eGFR after COVID-19 and pneumonia compared with the intercept from our fully adjusted models, with a 95% CI for each obtained using the delta method. We assessed for departure from linearity in eGFR decline by comparing our model with a model fitted with a quadratic term for time.

We then used cause-specific Cox proportional hazards regression to model the hazards of 25% reduction in eGFR after COVID-19 compared with pneumonia, adjusting for all covariates. We accounted for clustering by individuals using robust SEs.

Hypothesizing that more severe infections may have a steeper subsequent eGFR slope, we analyzed all outcomes after stratification by hospitalization status. We performed 3 sensitivity analyses excluding individuals without creatinine measurements both before and after infection, those who died during follow-up, and those with any prior history of pneumonia, respectively (eAppendix 4 in Supplement 1).

Data management was undertaken using Stata, version 16.1 (StataCorp LLC). Statistical analysis was performed between June 2023 and October 2024 using Stata, version 16.1 and R (R Project for Statistical Computing).

## Results

After applying inclusion and exclusion criteria, we included 134 565 individuals with COVID-19 and 35 987 individuals with pneumonia (eFigure 3 in Supplement 1). The COVID-19 cohort consisted of 74 819 females (55.6%) and 59 746 males (44.4%), with a median (IQR) age of 51 (37-64) years. The pneumonia cohort included 19 359 females (53.8%) and 16 628 males (46.2%), with a median (IQR) age of 71 (56-81) years (Table 1). The median (IQR) baseline eGFR was 94 (79-107) mL/min/1.73m^2^ for the COVID-19 cohort and 79 (61-92) mL/min/1.73m^2^ for the pneumonia cohort.

**Table 1.** Baseline Characteristics of Individuals With Incident COVID-19 and Pneumonia

Characteristic	Patients, No. (%)	
	With COVID-19	With Pneumonia
Number of included cases	134 565	35 987
Age, median (IQR), y	51 (37-64)	71 (56-81)
Sex		
Female	74 819 (55.6)	19 359 (53.8)
Male	59 746 (44.4)	16 628 (46.2)
Educational level		
Compulsory school	20 752 (15.4)	8207 (22.8)
Secondary school	49 009 (36.4)	14 161 (39.4)
University	51 292 (38.1)	10 979 (30.5)
Missing data	13 512 (10.0)	2640 (7.3)
Annual income tertile		
Lowest third income	32 076 (23.8)	11 392 (31.7)
Middle third income	49 032 (36.4)	15 440 (42.9)
Highest third income	42 278 (31.4)	7352 (20.4)
Missing data	11 179 (8.3)	1803 (5.0)
Creatinine tests before index date, median (IQR), No.	2 (1-3)	3 (2-6)
Baseline eGFR, median (IQR), mL/min/1.73m^2^	94 (79-107)	79 (61-92)
eGFR category at index date		
≥105 mL/min/1.73m^2^	33 369 (24.8)	3339 (9.3)
90-104 mL/min/1.73m^2^	33 802 (25.1)	6285 (17.5)
60-89 mL/min/1.73m^2^	40 283 (29.9)	16 046 (44.6)
30-59 mL/min/1.73m^2^	8683 (6.5)	6855 (19.0)
15-29 mL/min/1.73m^2^	868 (0.6)	795 (2.2)
<15 mL/min/1.73m^2^	274 (0.2)	137 (0.4)
Hypertension	40 736 (30.3)	16 132 (44.8)
Diabetes	15 405 (11.4)	5218 (14.5)
Cardiovascular diseases	24 837 (18.5)	11 608 (32.3)
Cancer, nonhematological	11 666 (8.7)	5810 (16.1)
Immunosuppressive diseases	8148 (6.1)	3787 (10.5)
History of pneumonia	25 425 (18.9)	7435 (20.7)
History of AKI in the preceding 5 y	3510 (2.6)	3112 (8.6)
Kidney transplant	409 (0.3)	144 (0.4)
RASi use	31 763 (23.6)	11 762 (32.7)
Hospitalizations in the previous 5 y, median (IQR), No.	0 (0-2)	2 (1-6)

Abbreviations: AKI, acute kidney injury; eGFR, estimated glomerular filtration rate; RASi, renin-angiotensin system inhibitor.

Individuals who were excluded because they lacked creatinine testing were younger and had fewer comorbidities than those included (eTable 1 in Supplement 1). Overall, 16 749 individuals (46.5%) in the pneumonia cohort required hospitalization, compared with 17 871 individuals (13.3%) in the COVID-19 cohort. Among those who were hospitalized, there was a clear pattern of more males, lower educational level and annual income, and more comorbidity with lower baseline kidney function compared with those not hospitalized (eTable 2 in Supplement 1). Of those hospitalized, 19.0% (3391 of 17 871) with COVID-19 and 22.7% (3794 of 16 749) with pneumonia had concurrent AKI. The COVID-19 cohort had a median (IQR) of 2 (1-3) creatinine tests before infection compared with 3 (2-6) tests before infection for the pneumonia cohort (eFigure 4 in Supplement 1). A total of 5004 individuals with both infections were selected in both cohorts (representing 185 [3.7%] of the COVID-19 cohort, and 195 [13.9%] of the pneumonia cohort).

During a median (IQR) follow-up of 10.8 (8.4-13.2) months for the COVID-19 group, 2061 (1.5%) individuals died at 17 (95% CI, 16-19) per 1000 person-years. For the pneumonia cohort, the median (IQR) follow-up was 10.8 (4.8-19.2) months, and 6091 (16.9%) individuals died at 175 (95% CI, 170-179) per 1000 person-years.

### Mean eGFR Slopes Before vs After COVID-19 or Pneumonia

**Figure 1.**
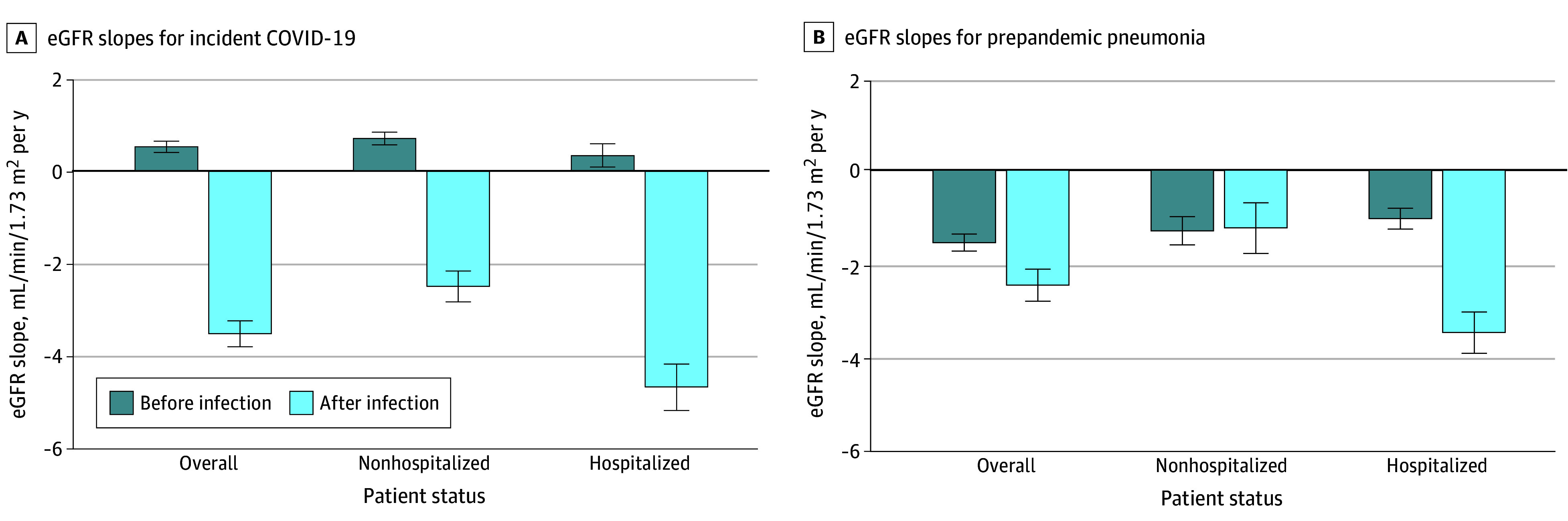
Age- and Sex-Adjusted Estimated Glomerular Filtration Rate (eGFR) Slopes Error bars indicate 95% CIs.

Before COVID-19, individuals had, on average, little change in eGFR, regardless of hospitalization status (Figure 1). After COVID-19, the mean decline in eGFR was 4.1 (95% CI, 3.8-4.4) mL/min/1.73m^2^ faster. eGFR decline was more pronounced after COVID-19 hospitalization than nonhospitalization (5.0 [95% CI, 4.5-5.6] mL/min/1.73m^2^ faster vs 3.2 [95% CI, 2.8-3.6] mL/min/1.73m^2^ faster; *P* for interaction < .001).

Before pneumonia, we observed a decline in eGFR (Figure 1). After pneumonia, the mean decline was 0.9 (95% CI, 0.5-1.3) mL/min/1.73m^2^ faster. The decline after pneumonia hospitalization accelerated by 2.4 (95% CI, 1.9-2.9) mL/min/1.73m^2^, but there was no evidence of accelerated decline after nonhospitalized pneumonia (0.1 [95% CI, −0.7 to 0.6] mL/min/1.73m^2^ faster; *P* for interaction < .001).

### Differences in Postinfection eGFR Slopes in COVID-19 vs Pneumonia

A total of 59 267 patients (44.0%) in the COVID-19 cohort and 20 138 (56.0%) patients in the pneumonia cohort had postinfection eGFR measurements and thereby composed the secondary dataset (eFigure 3 in Supplement 1). In general, these individuals were older and had more comorbidities than those without postinfection eGFR measurements (eTable 1 in Supplement 1). The median (IQR) number of creatinine tests available to estimate postinfection slopes was 2 (1-3) for the COVID-19 cohort and 2 (1-5) for the pneumonia cohort (eFigure 4 in Supplement 1).

**Figure 2.**
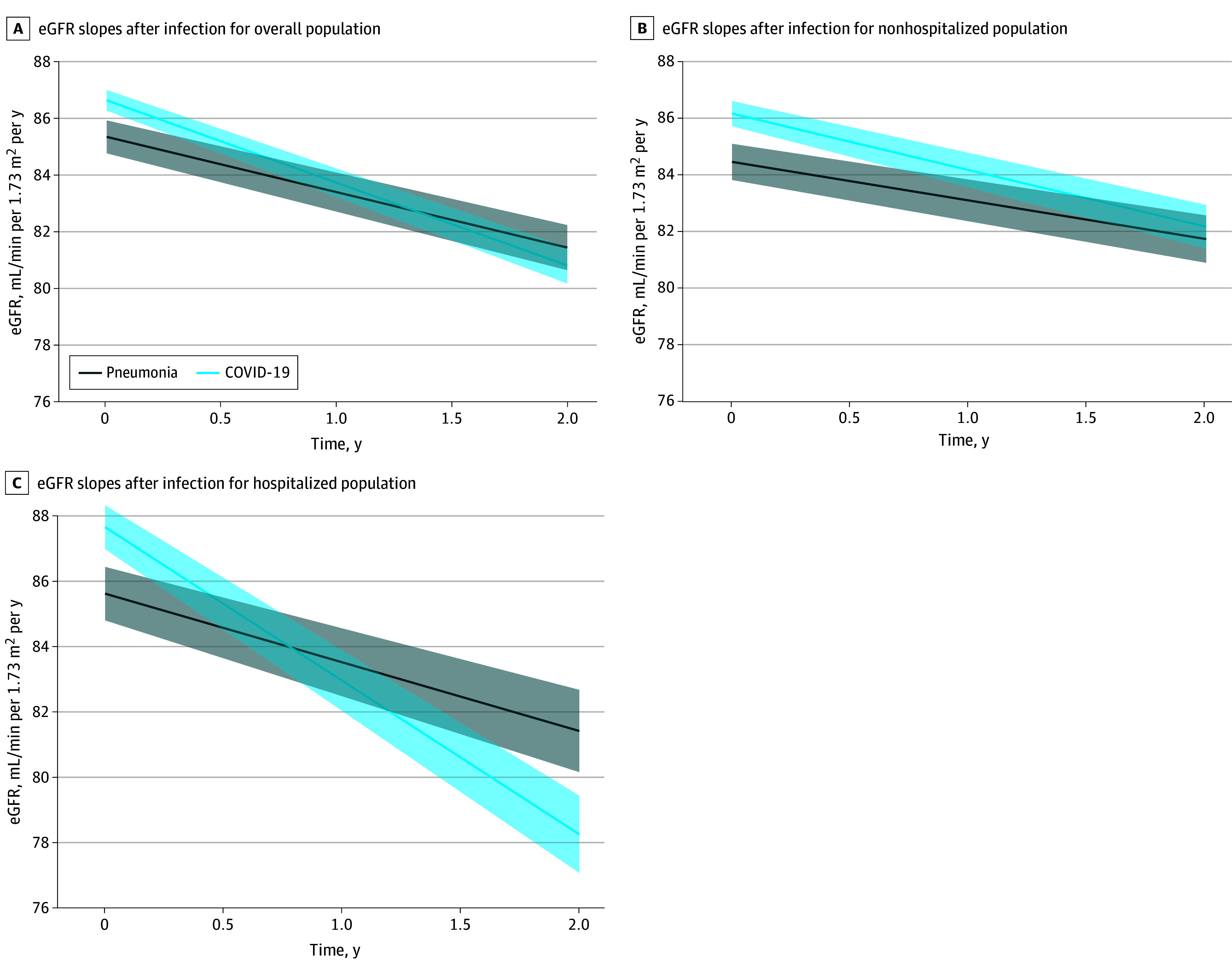
Estimated Glomerular Filtration Rate (eGFR) Slopes After COVID-19 vs Pneumonia Models were adjusted for age, sex, annual income, educational level, diabetes, hypertension, cardiovascular diseases, nonhematological cancer, immunosuppressed diseases, acute kidney injury, previous pneumonia, number of hospital admissions in the preceding 5 years, renin-angiotensin system inhibitor use in the preceding 6 months, eGFR slope before infection, baseline eGFR, and number of creatinine measurements before infection. Percentage decline in eGFR was calculated using delta method. Shaded area indicates 95% CIs.

After adjustment, the mean annual reduction in eGFR after COVID-19 was 3.4% (95% CI, 3.2%-3.5%), increasing to 5.4% (95% CI, 5.2%-5.6%) among those hospitalized (Figure 2). In the pneumonia group, the mean annual reduction in eGFR was 2.3% (95% CI, 2.1%-2.5%) and remained similar after restricting the analysis to those who were hospitalized. The COVID-19 group had an annual eGFR decline that was 1.0 (95% CI, 0.7-1.2) mL/min/1.73m^2^ faster than that for the pneumonia group. This difference was observed mainly among hospitalized cases; the COVID-19 group had an annual eGFR decline of 2.6 (95% CI, 2.3-3.0) mL/min/1.73m^2^ faster compared with the pneumonia group. The annual eGFR decline after nonhospitalized COVID-19 was 0.6 (95% CI, 0.3-1.0) mL/min/1.73m^2^ faster compared with after nonhospitalized pneumonia. We found no evidence of departure from linearity in eGFR decline.

### Reduction in eGFR After COVID-19 vs After Pneumonia

A 25% reduction in eGFR occurred in 1051 of 134 565 patients (0.8%) who survived COVID-19 compared with 619 of 35 987 patients (1.7%) who survived pneumonia. However, after adjustment for covariates, the hazard ratio (HR) for 25% reduction in eGFR was 1.19 (95% CI, 1.07-1.34) overall for COVID-19 vs pneumonia, increasing to 1.42 (95% CI, 1.22-1.64) among those who were hospitalized. Among those who were not hospitalized, there was no difference in 25% reduction in eGFR between COVID-19 and pneumonia (HR, 1.04; 95% CI, 0.88-1.24) (Table 2).

**Table 2.** Twenty-Five Percent Reduction in Estimated Glomerular Filtration Rate After COVID-19 vs Pneumonia, Overall and Stratified by Hospitalization Status^a^

Status	COVID-19		Pneumonia		COVID-19 vs pneumonia, HR (95% CI)
	Events, No. (%)	Crude rate (95% CI), per 1000 person-years	Events, No. (%)	Crude rate (95% CI), per 1000 person-years	
Overall	1051 (0.8)	162 (152-172)	619 (1.7)	248 (229-268)	1.19 (1.07-1.34)
Nonhospitalized	547 (0.5)	105 (97-115)	207 (1.1)	148 (129-169)	1.04 (0.88-1.24)
Hospitalized	504 (2.8)	385 (353-421)	412 (2.5)	374 (353-421)	1.42 (1.22-1.64)

Abbreviation: HR, hazard ratio.

^a^ Cause-specific Cox proportional hazards regression models were adjusted for age (as a cubic spline), sex, annual income, educational level, baseline estimated glomerular filtration rate, diabetes, hypertension, cardiovascular diseases, nonhematological cancer, immunosuppression diseases, previous acute kidney injury, previous pneumonia, number of hospital admissions in the preceding 5 years, renin-angiotensin system inhibitor use in the preceding 6 months, and number of creatinine tests in the preceding 2 years. Clustering by individual was accounted for using robust SEs.

### Sensitivity Analyses

Reanalyzing the primary outcome in the subset of participants with eGFR measurements after the infection (secondary dataset) provided results similar to the main analyses (eFigure 5 in Supplement 1). The results were also similar in analyses excluding individuals who died during follow-up (eFigures 6 and 7 in Supplement 1) and individuals with any prior history of pneumonia from either cohort (eFigures 8 and 9 in Supplement 1).

## Discussion

Using routinely collected, comprehensive health care data, we found an accelerated eGFR decline of a larger magnitude among survivors of COVID-19 than survivors of pneumonia due to other infections. We observed a steeper decline in eGFR for patients hospitalized for COVID-19 compared with patients hospitalized for pneumonia. These findings help inform decisions regarding the need to monitor kidney function in survivors of COVID-19 and could have implications for policymakers regarding future health care planning and kidney service provision.

We observed eGFR declines after COVID-19 that are comparable to data in previous reports.^7,8^ Some studies have compared kidney function outcomes in people with COVID-19 against contemporary noninfected controls, which may be challenging given that access to health care (including creatinine level monitoring) may have been limited.^7,10^ Comparison with noninfected general population controls also makes it difficult to assess whether findings are specific to COVID-19 (vs other serious infections).^7^ Recent studies have compared COVID-19 sequelae against patients hospitalized for influenza.^9,10^ The present study compared COVID-19 sequelae with those of pneumonia (including influenza) more broadly^20,21^ because restricting to seasonal influenza would have been more difficult to interpret given that outcomes may vary by strain, while also reducing sample size. In 1 study, a 25% or more decrease in eGFR was reduced after hospitalization with COVID-19–related AKI compared with other reasons for AKI hospitalization, including influenza.^10^ Conversely, there was greater annual decline in eGFR after COVID-19–related AKI compared with other AKIs and influenza-related AKI, highlighting the difficulty in interpreting findings in survivors of AKI.

The pre–COVID-19 eGFR slopes (which were slightly positive or >0) may be attributable to the younger and healthier COVID-19 cohort, who also had a higher baseline eGFR than the pneumonia cohort; creatinine-derived eGFR is an imperfect surrogate of true kidney function and is especially susceptible to fluctuation and measurement error in individuals with preserved kidney function.^22^ The most important finding of our study was the steeper eGFR decline in hospitalized COVID-19 cases compared with both preinfection eGFR slopes and hospitalized prepandemic pneumonia cases. It is possible that AKI during hospitalization explains this subsequent acceleration of eGFR decline, although investigating this was beyond the scope of our analysis because of differential survivor bias after AKI.^3,23,24^ The differences between COVID-19 and pneumonia may also be explained by reduced health care resources during the pandemic. Because our study concluded in January 2022, we had insufficient follow-up after health care services had recovered, and we recommend investigation over a longer period.

Our study offers additional novel perspectives. The retrospective evaluation of eGFR slopes from observational data requires that patients undergo creatinine testing (eAppendix 5 in Supplement 1).^15,25^ Patients without creatinine testing prior to infection were younger and less comorbid (ie, they likely did not have an indication for creatinine testing), and consequently we are unable to generalize the findings to these individuals. The proportion of patients excluded was larger for the COVID-19 cohort than for the pneumonia cohort, which we attribute to the magnitude of the COVID-19 pandemic; differences in virulence characteristics between pathogens; and selection criteria, given that individuals with pneumonia required a clinical diagnosis and those with COVID-19 were identified by a positive test result regardless of progression to respiratory tract disease. Furthermore, approximately half of both cohorts underwent creatinine testing before infection but never received monitoring for creatinine level afterward. The similarity in preinfection eGFR slopes regardless of postinfection testing leads us to speculate that similar acceleration in eGFR declines could have also occurred in those with only preinfection tests. People with pneumonia with postinfection creatinine tests were older and had greater comorbidity and therefore were more prone to eGFR decline compared with their counterparts in the COVID-19 cohort. If this difference imposed residual confounding, it would likely mean that we have underestimated the magnitude of faster eGFR decline after COVID-19. Additionally, we addressed the possibility of informative censoring by confirming the findings across participants who survived the whole follow up.

Generalizing these findings should be done with caution. We recommend triangulation with similar analyses in other health care systems and longer follow-up.

### Strengths and Limitations

Other strengths of this study include the capture of all positive COVID-19 and creatinine test results for an entire region with universal health care. Exclusion of eGFR tests 1 month before and 2 months after either COVID-19 or pneumonia minimized the association of acute illness with creatinine (eg, due to AKI or body composition changes),^26^ allowing for better estimation of baseline kidney function and the starting point of postinfection eGFR slopes.

The limitations of this study include those inherent in any observational study design as well as the lack of information on important confounders, such as ethnicity and body mass index. Although we were able to evaluate eGFR declines with a longer follow-up than most previous reports, we recognize that the follow-up is still too short to fully evaluate the long-term association of COVID-19 with kidney function. Moreover, some individuals may have been misclassified as nonhospitalized if their first infection was mild and a subsequent infection required hospitalization. This misclassification may explain the slight increase in eGFR decline after COVID-19 nonhospitalization compared with pneumonia nonhospitalization.

## Conclusions

In this cohort study, we found accelerated kidney function decline after severe COVID-19 that was of greater magnitude than after other causes of pneumonia. We therefore propose that people who were hospitalized for COVID-19 receive closer monitoring of kidney function to ensure prompt diagnosis and optimized management of chronic kidney disease to effectively prevent complications and further decline.^27^

## Notes

Supplement 1. **eFigure 1.** Schematic of Study Design **eFigure 2.** Directed Acyclic Graph to Describe Paths Between COVID-19 and Estimated Glomerular Filtration Rate (eGFR) Decline **eFigure 3.** Flowcharts for Selection of COVID-19 and Pneumonia Cohorts **eFigure 4.** Frequency of Creatinine Measurements Before and After COVID-19 Infection Pneumonia **eFigure 5.** Sensitivity Analysis (Only Cases With Post-Infection eGFR): Age- and Sex-Adjusted Estimated Glomerular Filtration Rate (eGFR) Slopes Before and After Incident COVID-19 Infection (Panel A) and Pre-Pandemic Pneumonia (Panel B), Overall and Stratified by Need for Hospitalisation **eFigure 6.** Sensitivity Analysis (Only Cases Alive During All Follow-up): Age- and Sex-Adjusted Estimated Glomerular Filtration Rate (eGFR) Slopes Before and After Incident COVID-19 Infection (Panel A) and Pre-Pandemic Pneumonia (Panel B), Overall and Stratified by Need for Hospitalisation **eFigure 7.** Sensitivity Analysis (Only Cases Alive During All Follow-up): Fully-Adjusted Estimated Glomerular Filtration Rate (eGFR) Slopes After COVID-19 or Pneumonia, Overall (Panel A) and Stratified by Need for Hospitalisation (Panel B and C) **eFigure 8.** Sensitivity Analysis (Excluding Anyone With Prior History of Pneumonia): Age- and Sex-Adjusted Estimated Glomerular Filtration Rate (eGFR) Slopes Before and After Incident COVID-19 Infection (Panel A) and Pre-Pandemic Pneumonia (Panel B), Overall and Stratified by Need for Hospitalisation **eFigure 9.** Sensitivity Analysis (Excluding Individuals With History of Pneumonia): Fully-Adjusted Estimated Glomerular Filtration Rate (eGFR) Slopes After COVID-19 or Pneumonia, Overall (Panel A) and Stratified by Need for Hospitalisation (Panel B and C) **eTable 1.** Characteristics of Included and Excluded Individuals Into Study Analyses Because on the Availability of Creatinine Tests Before and/or After the Infection Event **eTable 2.** Baseline Characteristics of Patients With COVID-19 and Pneumonia Stratified by Need for Hospitalisation **eAppendix 1.** Data Source: Stockholm Creatinine Measurements (SCREAM) Project **eAppendix 2.** International Classification of Disease Version 10 Codelists by Disease **eAppendix 3.** Details of Statistical Model **eAppendix 4.** Sensitivity Analyses **eAppendix 5.** Potential Sources of Bias

Supplement 2.
Data Sharing Statement
