# Supplementary material for: Kidney Function Decline After COVID-19 Infection
Source: JAMA Netw Open. 2024 Dec 26;7(12):e2450014. doi: 10.1001/jamanetworkopen.2024.50014 (PMC11672154; doi:10.1001/jamanetworkopen.2024.50014)
Supplement: Supplement 1. — eFigure 1. Schematic of Study Design eFigure 2. Directed Acyclic Graph to Describe Paths Between COVID-19 and Estimated Glomerular Filtration Rate (eGFR) Decline eFigure 3. Flowcharts for Selection of COVID-19 and Pneumonia Cohorts eFigure 4. Frequency of Creatinine Measurements Before and After COVID-19 Infection Pneumonia eFigure 5. Sensitivity Analysis (Only Cases With Post-Infection eGFR): Age- and Sex-Adjusted Estimated Glomerular Filtration Rate (eGFR) Slopes Before and After Incident COVID-19 Infection (Panel A) and Pre-Pandemic Pneumonia (Panel B), Overall and Stratified by Need for Hospitalisation eFigure 6. Sensitivity Analysis (Only Cases Alive During All Follow-up): Age- and Sex-Adjusted Estimated Glomerular Filtration Rate (eGFR) Slopes Before and After Incident COVID-19 Infection (Panel A) and Pre-Pandemic Pneumonia (Panel B), Overall and Stratified by Need for Hospitalisation eFigure 7. Sensitivity Analysis (Only Cases Alive During All Follow-up): Fully-Adjusted Estimated Glomerular Filtration Rate (eGFR) Slopes After COVID-19 or Pneumonia, Overall (Panel A) and Stratified by Need for Hospitalisation (Panel B and C) eFigure 8. Sensitivity Analysis (Excluding Anyone With Prior History of Pneumonia): Age- and Sex-Adjusted Estimated Glomerular Filtration Rate (eGFR) Slopes Before and After Incident COVID-19 Infection (Panel A) and Pre-Pandemic Pneumonia (Panel B), Overall and Stratified by Need for Hospitalisation eFigure 9. Sensitivity Analysis (Excluding Individuals With History of Pneumonia): Fully-Adjusted Estimated Glomerular Filtration Rate (eGFR) Slopes After COVID-19 or Pneumonia, Overall (Panel A) and Stratified by Need for Hospitalisation (Panel B and C) eTable 1. Characteristics of Included and Excluded Individuals Into Study Analyses Because on the Availability of Creatinine Tests Before and/or After the Infection Event eTable 2. Baseline Characteristics of Patients With COVID-19 and Pneumonia Stratified by Need for Hospitalisation eAppendix [file jamanetwopen-e2450014-s001.pdf]

Supplemental Online Content

Mahalingasivam V, Faucon A-L, Sjölander A, et al. Kidney function decline after COVID-19 infection. *JAMA Netw Open*. 2024;7(12):e2450014. doi:10.1001/jamanetworkopen.2024.50014

eFigure 1. Schematic of Study Design
eFigure 2. Directed Acyclic Graph to Describe Paths Between COVID-19 and Estimated Glomerular Filtration Rate (eGFR) Decline
eFigure 3. Flowcharts for Selection of COVID-19 and Pneumonia Cohorts
eFigure 4. Frequency of Creatinine Measurements Before and After COVID-19 Infection Pneumonia
eFigure 5. Sensitivity Analysis (Only Cases With Post-Infection eGFR): Age- and Sex-Adjusted Estimated Glomerular Filtration Rate (eGFR) Slopes Before and After Incident COVID-19 Infection (Panel A) and Pre-Pandemic Pneumonia (Panel B), Overall and Stratified by Need for Hospitalisation
eFigure 6. Sensitivity Analysis (Only Cases Alive During All Follow-up): Age- and Sex-Adjusted Estimated Glomerular Filtration Rate (eGFR) Slopes Before and After Incident COVID-19 Infection (Panel A) and Pre-Pandemic Pneumonia (Panel B), Overall and Stratified by Need for Hospitalisation
eFigure 7. Sensitivity Analysis (Only Cases Alive During All Follow-up): Fully-Adjusted Estimated Glomerular Filtration Rate (eGFR) Slopes After COVID-19 or Pneumonia, Overall (Panel A) and Stratified by Need for Hospitalisation (Panel B and C)
eFigure 8. Sensitivity Analysis (Excluding Anyone With Prior History of Pneumonia): Age- and Sex-Adjusted Estimated Glomerular Filtration Rate (eGFR) Slopes Before and After Incident COVID-19 Infection (Panel A) and Pre-Pandemic Pneumonia (Panel B), Overall and Stratified by Need for Hospitalisation
eFigure 9. Sensitivity Analysis (Excluding Individuals With History of Pneumonia): Fully-Adjusted Estimated Glomerular Filtration Rate (eGFR) Slopes After COVID-19 or Pneumonia, Overall (Panel A) and Stratified by Need for Hospitalisation (Panel B and C)
eTable 1. Characteristics of Included and Excluded Individuals Into Study Analyses Because on the Availability of Creatinine Tests Before and/or After the Infection Event
eTable 2. Baseline Characteristics of Patients With COVID-19 and Pneumonia Stratified by Need for Hospitalisation
eAppendix 1. Data Source: Stockholm Creatinine Measurements (SCREAM) Project
eAppendix 2. International Classification of Disease Version 10 Codelists by Disease
eAppendix 3. Details of Statistical Model
eAppendix 4. Sensitivity Analyses
eAppendix 5. Potential Sources of Bias

This supplemental material has been provided by the authors to give readers additional information about their work.

© 2024 Mahalingasivam V et al. *JAMA Network Open*

eFigure 1. Schematic of Study Design

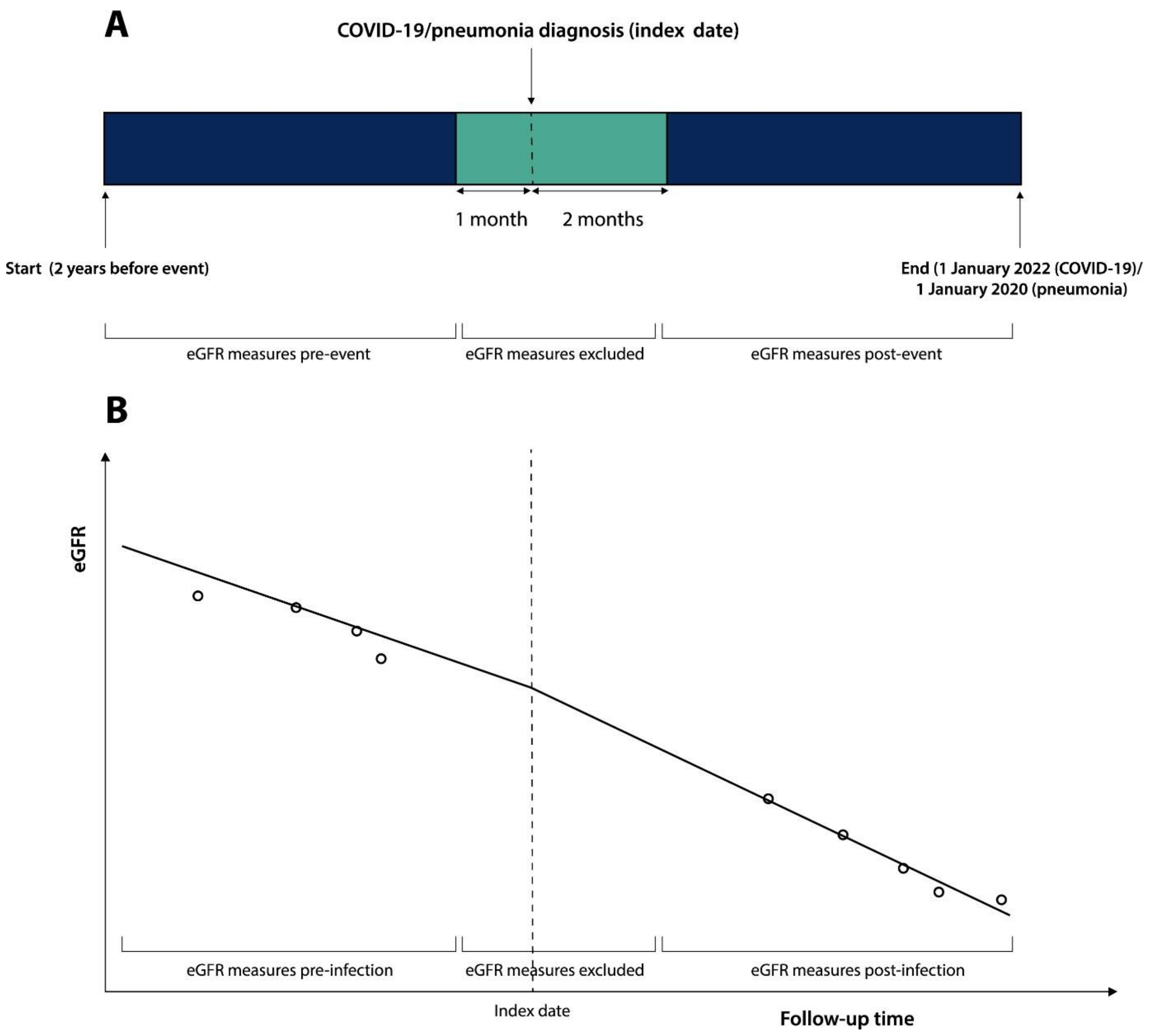

Panel A – The index date is defined as the date of COVID-19 diagnosis between February 2020 and January 2022, or the date of pneumonia diagnosis between February 2018 and January 2020. Estimated glomerular filtration rate (eGFR) measurements one month before and two months after the index date are excluded from analysis. All other outpatient eGFR measurements two years before the index date and up to either January 2022 for COVID-19 or January 2020 for pneumonia are included.

Panel B – Circles denote example valid outpatient eGFR measurements used to estimate slopes before the index date (pre-infection) and after the index date (post-infection).

© 2024 Mahalingasivam V et al. *JAMA Network Open*

eFigure 2. Directed Acyclic Graph to Describe Paths Between COVID-19 and Estimated Glomerular Filtration Rate (eGFR) Decline

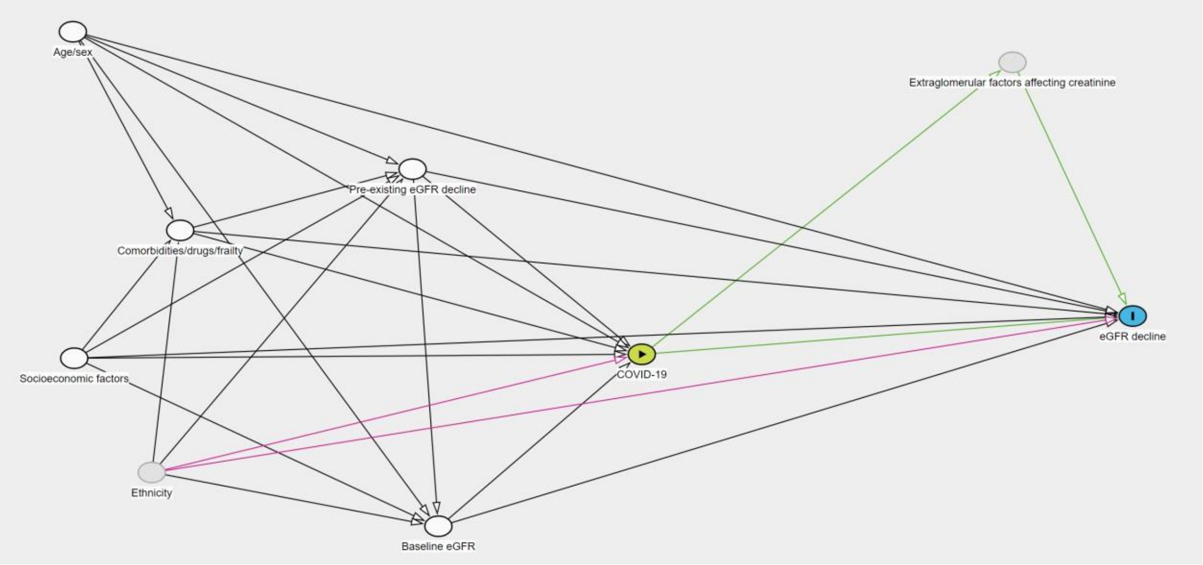

We used this model to select the confounders of our study: age, sex, socioeconomic factors, selected comorbidities and pre-existing eGFR decline. There may be residual confounding due to ethnicity or unmeasured clinical factors. COVID-19 may also have effects on body composition leading to extraglomerular reduction in serum creatinine and consequently overestimation of eGFR.

© 2024 Mahalingasivam V et al. *JAMA Network Open*

eFigure 3. Flowcharts for Selection of COVID-19 and Pneumonia Cohorts

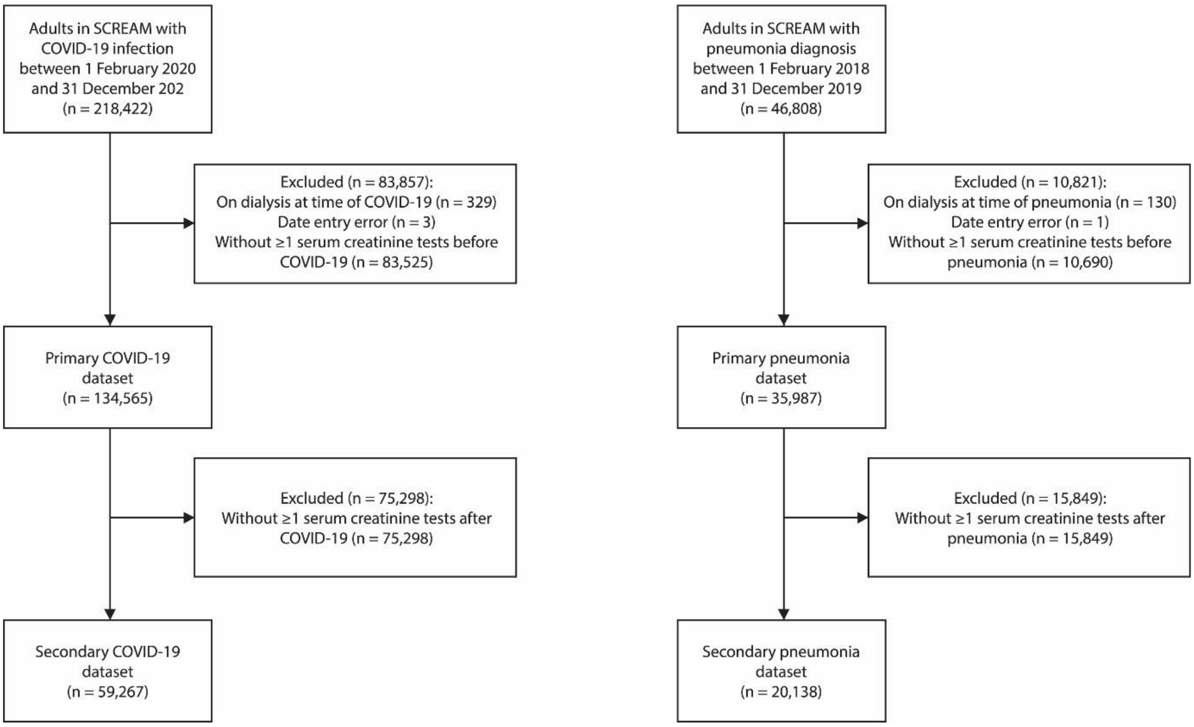

© 2024 Mahalingasivam V et al. *JAMA Network Open*

eFigure 4. Frequency of Creatinine Measurements Before and After COVID-19 Infection Pneumonia

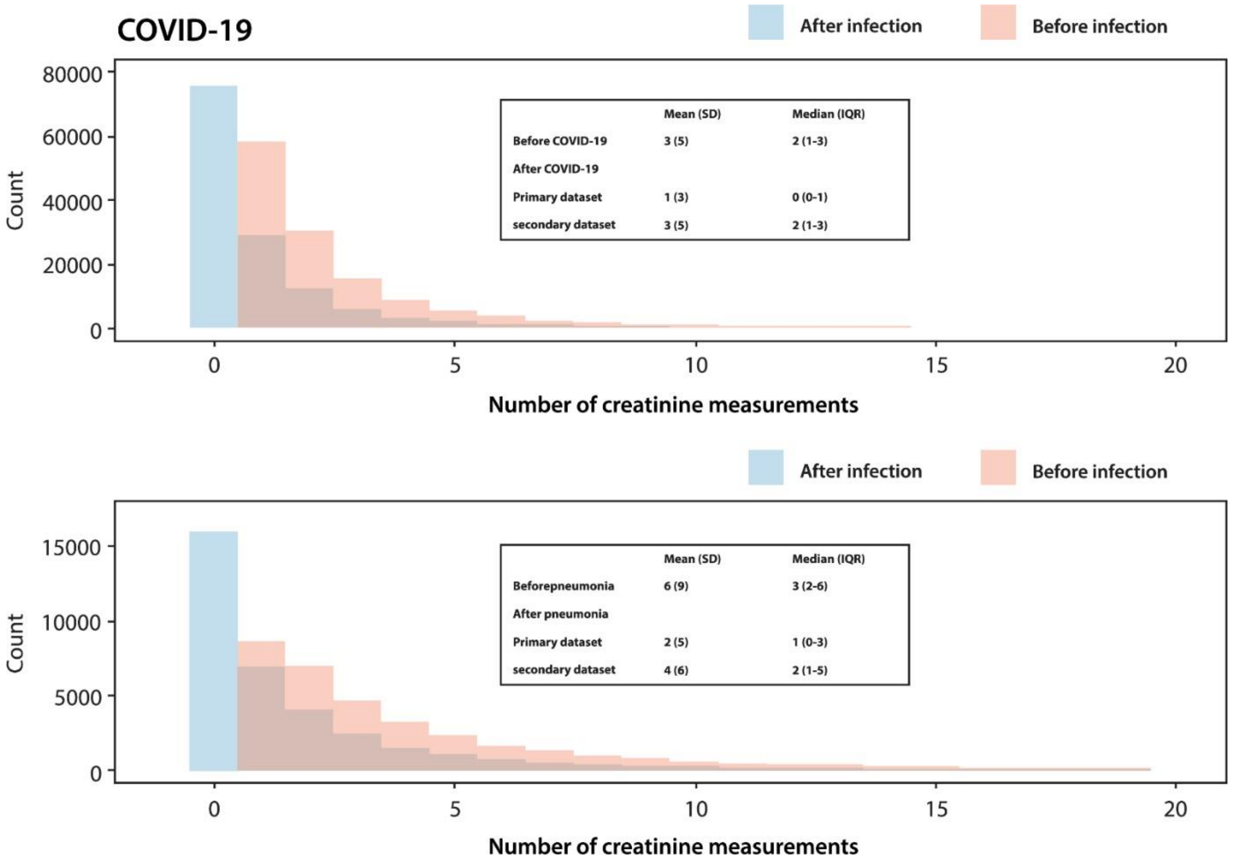

Primary dataset = individuals with at least one creatinine measurement before infection, secondary dataset = individuals with at least one creatinine measurement before and after infection, SD = standard deviation, IQR = interquartile range

© 2024 Mahalingasivam V et al. *JAMA Network Open*

eFigure 5. Sensitivity Analysis (Only Cases With Post-Infection eGFR): Age- and Sex-Adjusted Estimated Glomerular Filtration Rate (eGFR) Slopes Before and After Incident COVID-19 Infection (Panel A) and Pre-Pandemic Pneumonia (Panel B), Overall and Stratified by Need for Hospitalisation

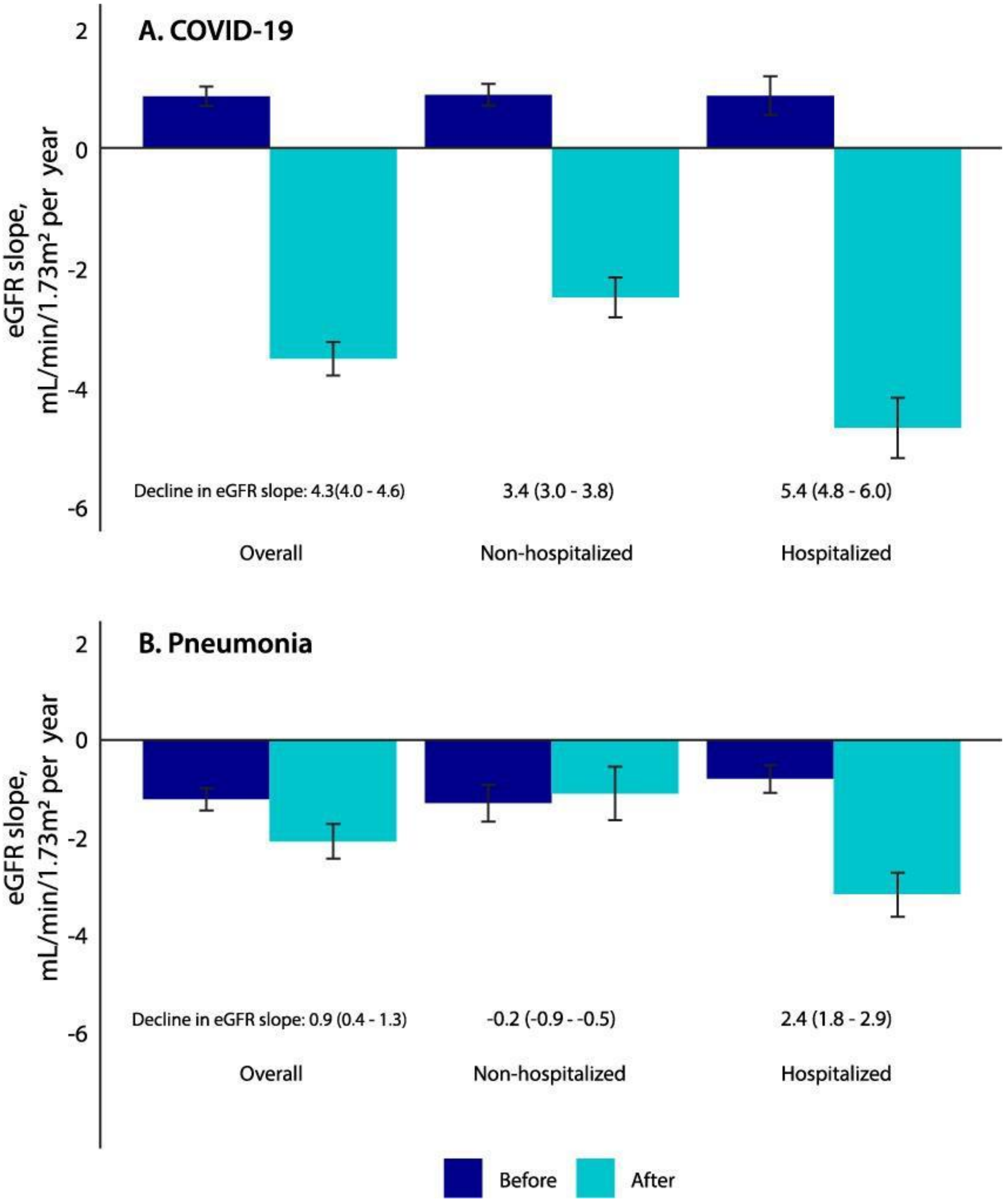

Analysis restricted to individuals with creatinine measurements after the infection (n= 59267 in the COVID-19 cohort and n= 20138 in the pneumonia cohort). Differences in eGFR slope are shown in ml/min/1.73m ^{2}/year.

© 2024 Mahalingasivam V et al. *JAMA Network Open*

eFigure 6. Sensitivity Analysis (Only Cases Alive During All Follow-up): Age- and Sex-Adjusted Estimated Glomerular Filtration Rate (eGFR) Slopes Before and After Incident COVID-19 Infection (Panel A) and Pre-Pandemic Pneumonia (Panel B), Overall and Stratified by Need for Hospitalisation

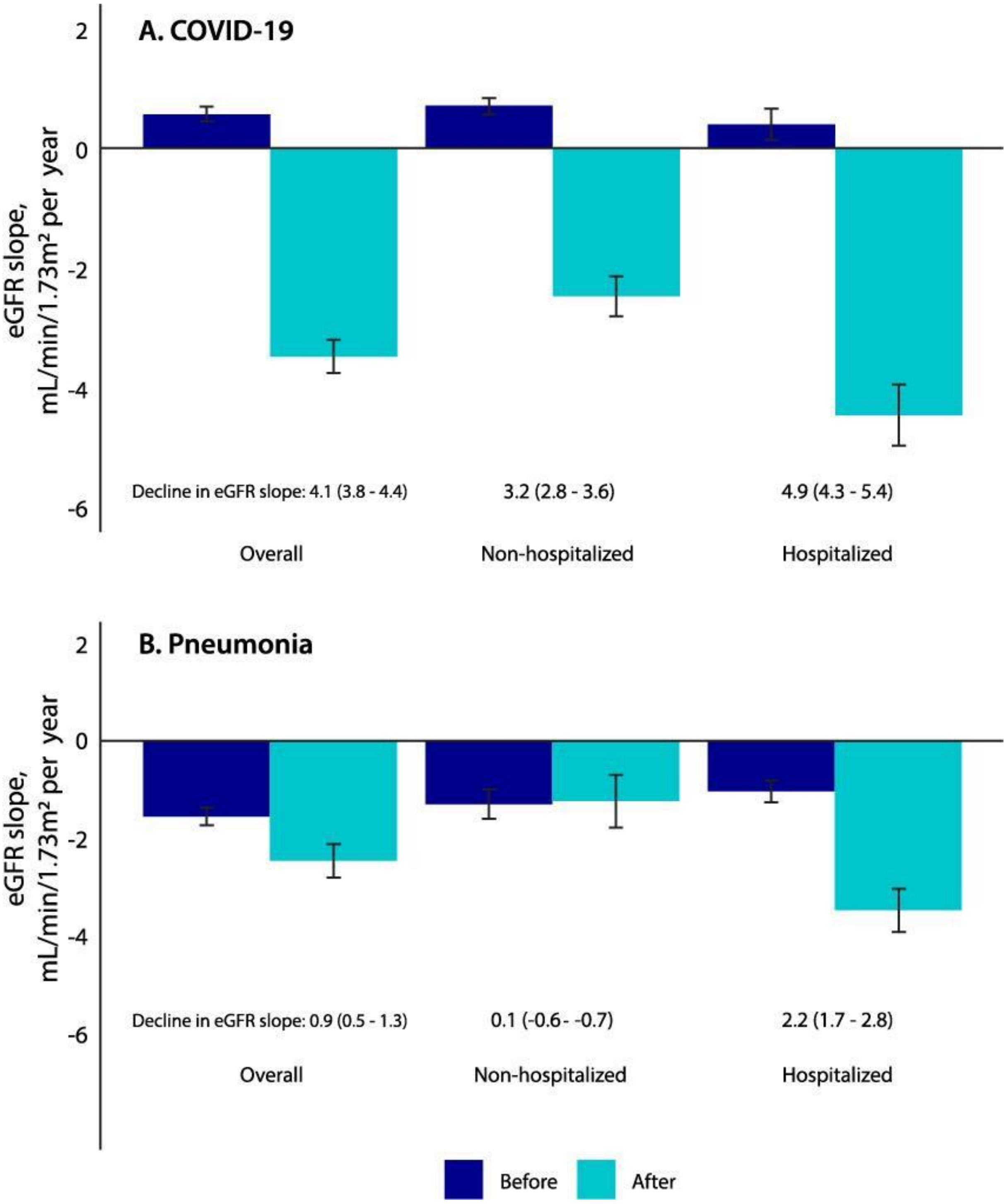

Analysis restricted to individuals who were alive until the end of follow-up (n= 132,504 in the COVID-19 cohort and n= 29.896 in the pneumonia cohort). Differences in eGFR slope are shown in ml/min/1.73m ^{2}/year.

© 2024 Mahalingasivam V et al. *JAMA Network Open*

eFigure 7

Sensitivity Analysis (Only Cases Alive During All Follow-up): Fully-Adjusted Estimated Glomerular Filtration Rate (eGFR) Slopes After COVID-19 or Pneumonia, Overall (Panel A) and Stratified by Need for Hospitalisation (Panel B and C)

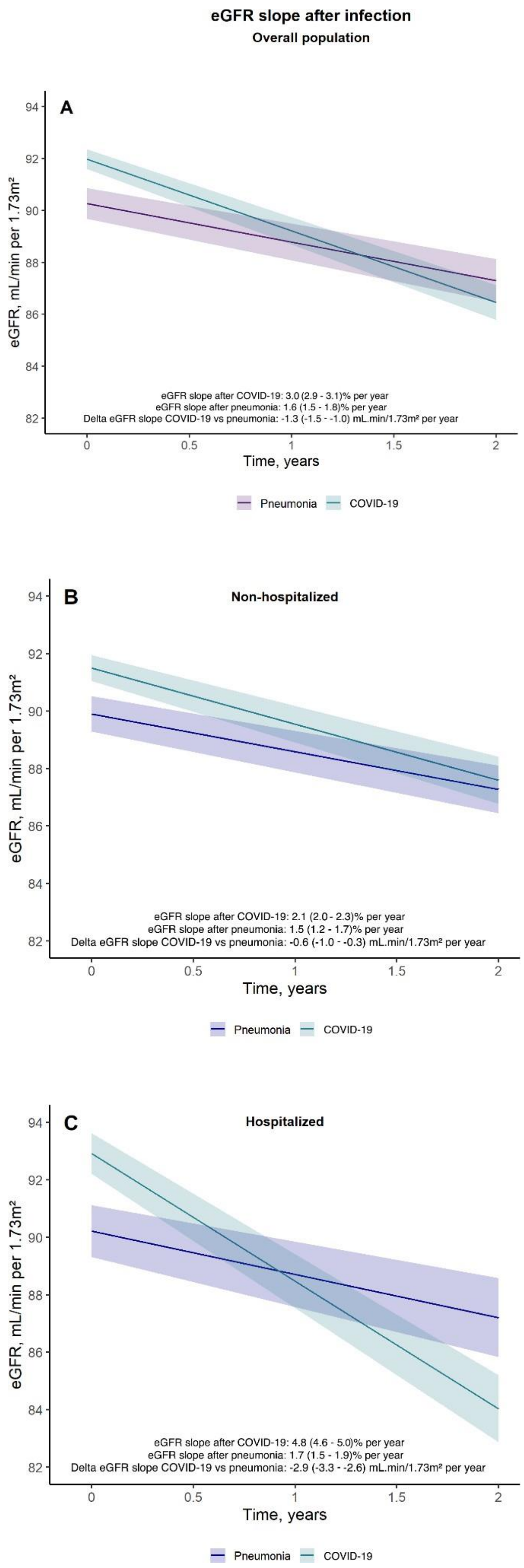

Analysis restricted to individuals who were alive until the end of follow-up (n= 59,267 in the COVID-19 cohort and n= 20,138 in the pneumonia cohort). Differences in eGFR slope are shown in ml/min/1.73m^2/year. Models adjusted for age, sex, income, education, diabetes, hypertension, cardiovascular disease, non-haematological cancer, immunosuppressed state, AKI, previous pneumonia, number of hospital admissions in the preceding 5 years, RASi use in the preceding 6 months, eGFR slope before infection, baseline eGFR, and number of creatinine measurements prior to infection.

© 2024 Mahalingasivam V et al. *JAMA Network Open*

eFigure 8. Sensitivity Analysis (Excluding Anyone With Prior History of Pneumonia): Age- and Sex-Adjusted Estimated Glomerular Filtration Rate (eGFR) Slopes Before and After Incident COVID-19 Infection (Panel A) and Pre-Pandemic Pneumonia (Panel B), Overall and Stratified by Need for Hospitalisation

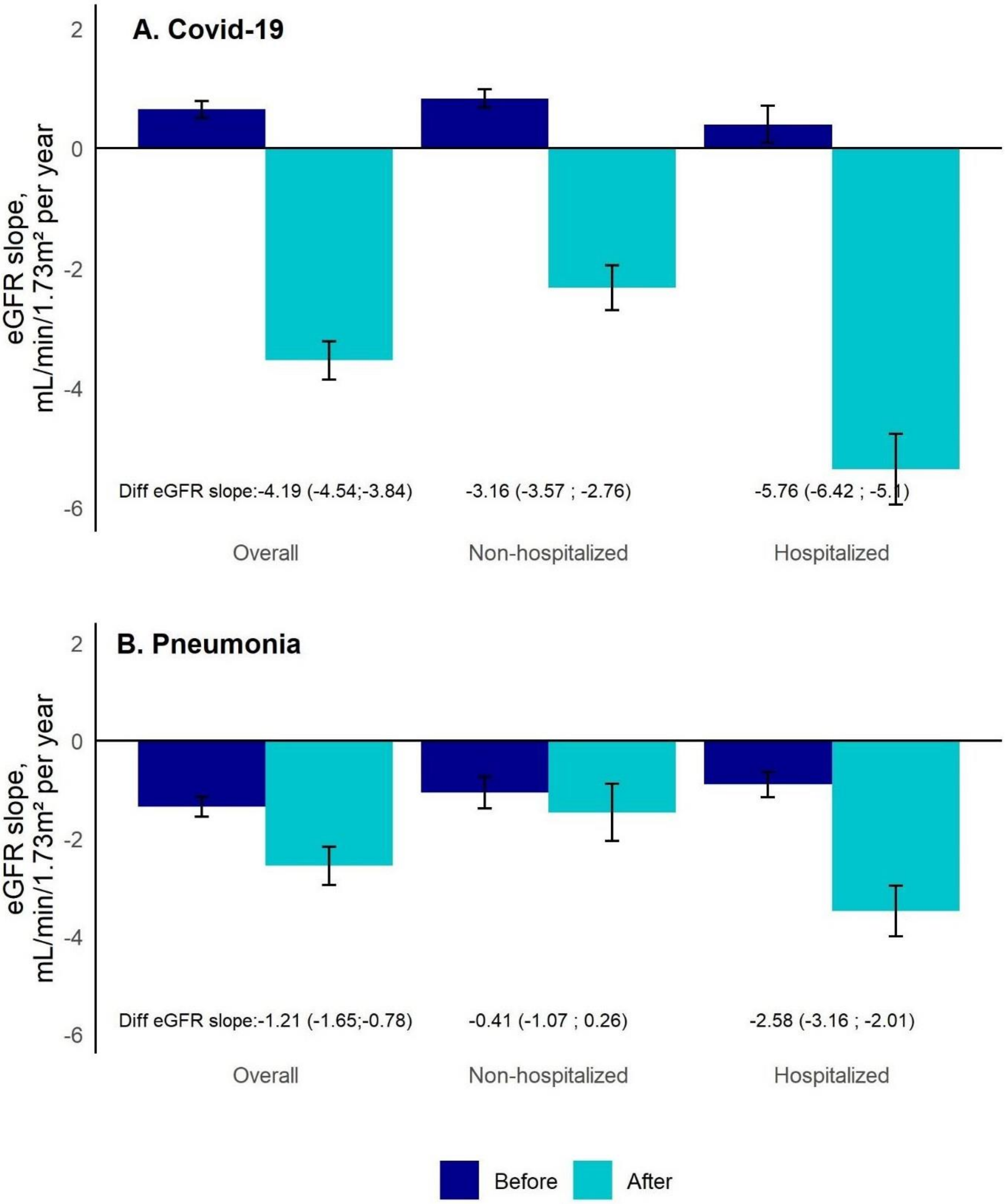

Analysis restricted to individuals without prior history of pneumonia (n=109,140 in the COVID-19 cohort and n=28,552 in the pneumonia cohort). Differences in eGFR slope are shown in ml/min/1.73m ^{2}/year.

© 2024 Mahalingasivam V et al. *JAMA Network Open*

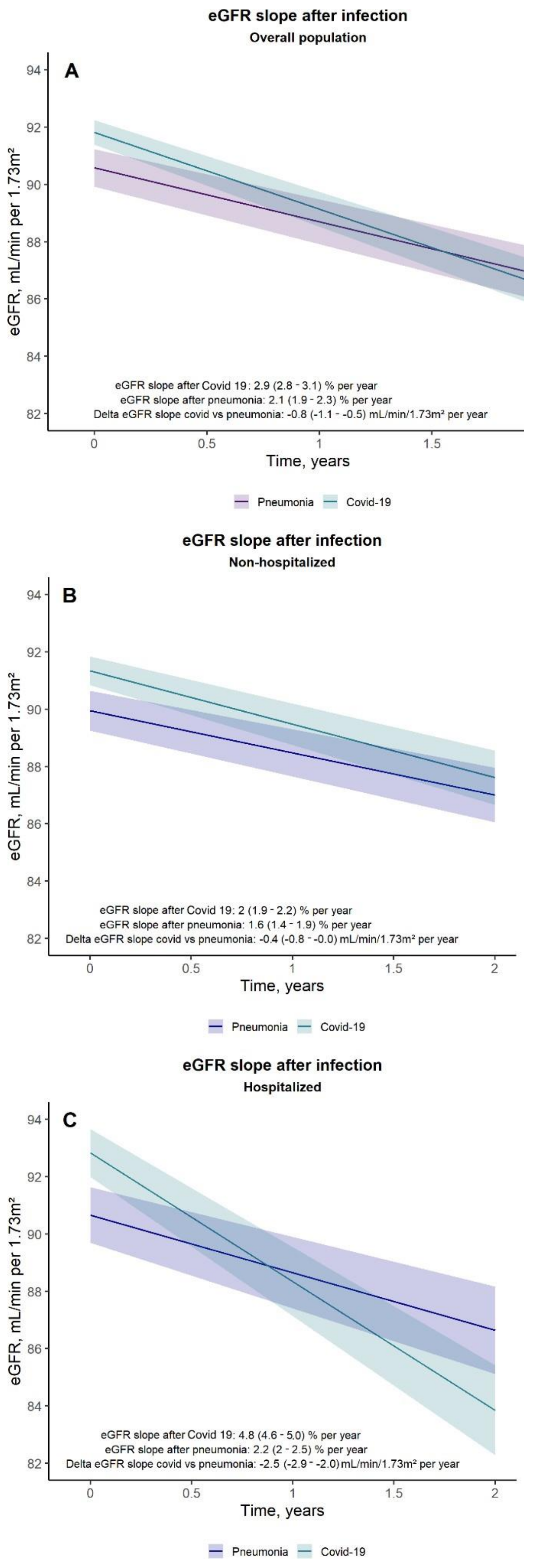

eFigure 9

Sensitivity Analysis (Excluding Individuals With History of Pneumonia): Fully-Adjusted Estimated Glomerular Filtration Rate (eGFR) Slopes After COVID-19 or Pneumonia, Overall (Panel A) and Stratified by Need for Hospitalisation (Panel B and C)

Analysis restricted to individuals without history of pneumonia and creatinine measurements both before and after infection (n=46,229 in the COVID-19 cohort and n= 15,733 in the pneumonia cohort). Differences in eGFR slope are shown in ml/min/1.73m^2/year. Models adjusted for age, sex, income, education, diabetes, hypertension, cardiovascular disease, non-haematological cancer, immunosuppressed state, AKI, number of hospital admissions in the preceding 5 years, RASi use in the preceding 6 months, eGFR slope before infection, baseline eGFR, and number of creatinine measurements prior to infection.

© 2024 Mahalingasivam V et al. *JAMA Network Open*

eTable 1. Characteristics of Included and Excluded Individuals Into Study Analyses Because on the Availability of Creatinine Tests Before and/or After the Infection Event

	COVID-19			Pneumonia		
	eGFR tested both before and after infection	eGFR tested ONLY before infection	Missing eGFR	eGFR tested both before and after infection	eGFR tested ONLY before infection	Missing eGFR
Number of cases	59267	75298	84186	20138	15849	10690
Age (years), Median (IQR)	56 (44-71)	46 (34-59)	39 (30-49)	72 (59-81)	70 (52-82)	45 (34-58)
Sex						
Female, n (%)	32798 (55.3)	42021 (55.8)	47548 (56.5)	10838 (53.8)	8521 (53.8)	5673 (53.1)
Male, n (%)	26469 (44.7)	33277 (44.2)	36638 (43.5)	9300 (46.2)	7328 (46.2)	5017 (46.9)
Educational level, n (%)						
Compulsory school	10282 (17.3)	10470 (13.9)	9430 (11.2)	4652 (23.1)	3555 (22.4)	1303 (12.2)
Secondary school	22355 (37.7)	26654 (35.4)	28080 (33.4)	8036 (39.9)	6125 (38.6)	3867 (36.2)
University	21098 (35.6)	30194 (40.1)	38248 (45.4)	6035 (30.0)	4944 (31.2)	4605 (43.1)
Missing	5532 (9.3)	7980 (10.6)	8428 (10.0)	1415 (7.0)	1225 (7.7)	915 (8.6)
Annual income tertile, n (%)						
Lowest third income	14834 (25.0)	17242 (22.9)	17870 (21.2)	6418 (31.9)	4974 (31.4)	2230 (20.9)
Middle third income	22490 (37.9)	26542 (35.2)	28645 (34.0)	8848 (43.9)	6592 (41.6)	3868 (36.2)
Highest third income	17366 (29.3)	24912 (33.1)	30127 (35.8)	3916 (19.4)	3436 (21.7)	3891 (36.4)
Missing	4577 (7.7)	6602 (8.8)	7544 (9.0)	956 (4.7)	847 (5.3)	701 (6.6)
Creatinine tests before index date, Median (IQR)	2 (1-4)	1 (1-2)	N/A	4 (2-7)	2 (1-5)	N/A
Baseline eGFR, Median (IQR)	90 (75-103)	97 (83-110)	N/A	77 (61-91)	80 (63-94)	N/A
eGFR category at index date, N (%)n						
≥105 ml/min/1.73m^2	11767 (19.9)	21602 (28.7)	N/A	1546 (7.7)	1793 (11.3)	N/A
90-104 ml/min/1.73m^2	15365 (25.9)	18437 (24.5)	N/A	3528 (17.5)	2757 (17.4)	N/A

© 2024 Mahalingasivam V et al. *JAMA Network Open*

60-89 ml/min/1.73m^2	21124 (35.6)	19159 (25.4)	N/A	9445 (46.9)	6601 (41.6)	N/A
30-59 ml/min/1.73m^2	5446 (9.2)	3237 (4.3)	N/A	4119 (20.5)	2736 (17.3)	N/A
15-29 ml/min/1.73m^2	524 (0.9)	344 (0.5)	N/A	441 (2.2)	354 (2.2)	N/A
<15 ml/min/1.73m^2	193 (0.3)	81 (0.1)	N/A	79 (0.4)	58 (0.4)	N/A
Hypertension, n (%)	22668 (38.2)	18068 (24.0)	3886 (4.6)	9018 (44.8)	7114 (44.9)	851 (8.0)
Diabetes mellitus, n (%)	9224 (15.6)	6181 (8.2)	1146 (1.4)	3115 (15.5)	2103 (13.3)	162 (1.5)
Cardiovascular diseases, n (%)	13676 (23.1)	11161 (14.8)	3289 (3.9)	6080 (30.2)	5528 (34.9)	545 (5.1)
Cancer (non-haematological), n (%)	6688 (11.3)	4978 (6.6)	1660 (2.0)	3254 (16.2)	2556 (16.1)	349 (3.3)
Immunosuppressive diseases, n (%)	4840 (8.2)	3308 (4.4)	1295 (1.5)	2273 (11.3)	1514 (9.6)	191 (1.8)
History of pneumonia, n (%)	13038 (22.0)	12387 (16.5)	9157 (10.9)	4405 (21.9)	3030 (19.1)	920 (8.6)
History of AKI in the preceding 5 years, n (%)	2264 (3.8)	1246 (1.7)	201 (0.2)	1919 (9.5)	1193 (7.5)	111 (1.0)
Kidney transplantation, n (%)	359 (0.6)	50 (0.1)	20 (0.0)	117 (0.6)	27 (0.2)	1 (0.0)
RASi use, n (%)	19125 (32.3)	12638 (16.8)	2526 (3.0)	7184 (35.7)	4578 (28.9)	560 (5.2)
Number of hospitalisation in the previous 5 years, Median (IQR)	1 (0-3)	0 (0-1)	0 (0-0)	3 (1-7)	2 (0-5)	0 (0-1)

AKI = acute kidney injury, eGFR = estimated glomerular filtration rate, IQR = interquartile range, RASi = renin-angiotensin system inhibition

© 2024 Mahalingasivam V et al. *JAMA Network Open*

eTable 2. Baseline Characteristics of Patients With COVID-19 and Pneumonia Stratified by Need for Hospitalisation

	COVID-19		Pneumonia	
	Non-hospitalised	Hospitalised	Non-hospitalised	Hospitalised
Number of included cases	116694	17871	19238	16749
Age (years), Median (IQR)	49 (36-61)	70 (57-81)	62 (48-74)	78 (69-86)
Sex				
Female, n (%)	66675 (57.1)	8144 (45.6)	11252 (58.5)	8107 (48.4)
Male, n (%)	50019 (42.9)	9727 (54.4)	7986 (41.5)	8642 (51.6)
Educational level, n (%)				
Compulsory school	16289 (14.0)	4463 (25.0)	3360 (17.5)	4847 (28.9)
Secondary school	42226 (36.2)	6783 (38.0)	7621 (39.6)	6540 (39.0)
University	46237 (39.6)	5055 (28.3)	6844 (35.6)	4135 (24.7)
Missing	11942 (10.2)	1570 (8.8)	1413 (7.3)	1227 (7.3)
Annual income tertile, n (%)				
Lowest third income	25773 (22.1)	6303 (35.3)	4822 (25.1)	6570 (39.2)
Middle third income	41744 (35.8)	7288 (40.8)	7939 (41.3)	7501 (44.8)
Highest third income	38943 (33.4)	3335 (18.7)	5366 (27.9)	1986 (11.9)
Missing	10234 (8.8)	945 (5.3)	1111 (5.8)	692 (4.1)
Creatinine tests before index date, Median (IQR)	2 (1-3)	3 (2-6)	2 (1-4)	4 (2-9)
Baseline eGFR, Median (IQR)	96 (82-109)	79 (61-94)	84 (70-97)	70 (53-86)
eGFR category at index date, n (%)				
≥105 ml/min/1.73m^2	31573 (27.1)	1796 (10.0)	2485 (12.9)	854 (5.1)
90-104 ml/min/1.73m^2	30384 (26.0)	3418 (19.1)	4187 (21.8)	2098 (12.5)
60-89 ml/min/1.73m^2	32804 (28.1)	7479 (41.8)	8538 (44.4)	7508 (44.8)
30-59 ml/min/1.73m^2	5336 (4.6)	3347 (18.7)	2090 (10.9)	4765 (28.4)
15-29 ml/min/1.73m^2	430 (0.4)	438 (2.5)	146 (0.8)	649 (3.9)
<15 ml/min/1.73m^2	112 (0.1)	162 (0.9)	18 (0.1)	119 (0.7)
Hypertension, n (%)	30656 (26.3)	10080 (56.4)	6486 (33.7)	9646 (57.6)
Diabetes mellitus, n (%)	11020 (9.4)	4385 (24.5)	1911 (9.9)	3307 (19.7)
Cardiovascular diseases, n (%)	17875 (15.3)	6962 (39.0)	4091 (21.3)	7517 (44.9)
Cancer (non-haematological), n (%)	8581 (7.4)	3085 (17.3)	2074 (10.8)	3736 (22.3)
Immunosuppressive diseases, n (%)	6157 (5.3)	1991 (11.1)	1446 (7.5)	2341 (14.0)
History of pneumonia, n (%)	20349 (17.4)	5076 (28.4)	2928 (15.2)	4507 (26.9)
History of AKI in the preceding 5 years, n (%)	1528 (1.3)	1982 (11.1)	539 (2.8)	2573 (15.4)
Kidney transplant, n (%)	278 (0.2)	131 (0.7)	42 (0.2)	102 (0.6)
RASi use, n (%)	24070 (20.6)	7693 (43.0)	5591 (29.1)	6171 (36.8)
Number of hospitalisation in the previous 5 years, Median (IQR)	0 (0-1)	3 (1-6)	1 (0-2)	6 (3-10)

AKI = acute kidney injury, eGFR = estimated glomerular filtration rate, IQR = interquartile range, RASi = renin-angiotensin system inhibition

© 2024 Mahalingasivam V et al. *JAMA Network Open*

eAppendix 1. Data Source: Stockholm Creatinine Measurements (SCREAM) Project

The Stockholm Creatinine Measurements (SCREAM) project is a complete and recurrent healthcare utilization data linkage from the region of Stockholm, Sweden, which at present has data up to 2022.] A single healthcare provider in the region provides universal, tax-funded healthcare to 20-25% of the population of Sweden. Using unique personal identification numbers, SCREAM linked regional and national administrative databases that hold complete information on demographics, socioeconomic data, healthcare utilization, laboratory tests results, dispensed drugs, diagnoses, and vital status without loss of follow-up.

The regional ethical review board in Stockholm approved the study (2017/793 -31); informed consent was not deemed necessary because data were deidentified at the Swedish Board of Health and Welfare.

eAppendix 2. International Classification of Disease Version 10 Codelists by Disease

COVID-19:
U07: COVID-19

Pneumonia:
B01.2: Chickenpox with pneumonia
B05.2: Pneumonia as a complication of measles
B20.6: HIV infection with Pneumocystis jirovecii (carinii)-pneumonia
B25.0: Cytomegalovirus pneumonitis
J09-J18: Influenza and pneumonia
J85.1: Pulmonary abscess with pneumonia
U04: Severe Acute Respiratory Syndrome [SARS]

Diabetes:
E10-E14: Diabetes
G59.0: Diabetic mononeuropathy
G63.2: Diabetic polyneuropathy
H28.0: Diabetic cataract
H36.0: Diabetic retinopathy
M14.2: Diabetic arthropathy
N08.3: Glomerular Diseases in Diabetes Mellitus
O24: Diabetes during pregnancy
Z96.4: Presence of endocrine implants

Hypertension:
H35: Other pathological changes in the retina
I10: Essential hypertension (high blood pressure with no known cause)
I15: Secondary hypertension (high blood pressure as a result of another disease)
I67.4: Hypertensive encephalopathy
Cardiovascular diseases:
I11: Hypertension with heart disease
I13: Hypertension with heart and kidney disease
I20-I25: Ischemic heart diseases (diseases caused by insufficient blood supply to the heart muscle)
I34: Non-rheumatic mitral valve diseases
I35: Non-rheumatic aortic valve diseases
I42: Cardiomyopathy (heart muscle disease)
I43: Myocardial disease in diseases classified elsewhere
I44: Atrioventricular block and left-sided branch block (conduction disorder between the atria and ventricle and in the left leg respectively)
I45: Other conduction disorders
I47: Paroxysmal tachycardia
I48: Atrial fibrillation and atrial flutter
I49: Other cardiac arrhythmias
I50: Cardiac insufficiency
I51.6: Cardiovascular Disease, Unspecified
I51.9: Heart Disease, Unspecified
I61: Stroke
I63: Cerebral infarction
I65: Occlusion and stenosis of the precerebral arteries not resulting in cerebral infarction
I66: Occlusion and stenosis of cerebral arteries not resulting in cerebral infarction
I67.2: Cerebral atherosclerosis
I67.8: Other specified cerebrovascular diseases
I67.9: Cerebrovascular Disease, Unspecified
I69.1: Late effects of intracerebral haemorrhage
I69.3: Late effects of cerebral infarction
I69.4: Late effects of cerebrovascular disease, not specified as bleeding or infarction
I69.8: Late effects of other and unspecified cerebrovascular diseases

© 2024 Mahalingasivam V et al. *JAMA Network Open*

I70: Atherosclerosis (hardening of the arteries)
I71: Aortic aneurysm
I72: Other aneurysm and dissection
I73.9: Peripheral Vascular Disease, Unspecified
I74: Embolism and thrombosis of the artery
I79.0: Aortic aneurysms in diseases classified elsewhere

Cancer (non-haematological):
C00-C14: Malignant tumors of the lip, oral cavity and pharynx
C15-C26: Malignant tumors of the digestive organs
C30-C39: Malignant tumors of the respiratory and thoracic organs
C40-C41: Malignant tumors of bone and articular cartilage
C43: Malignant melanoma of the skin
C45-C49: Malignant neoplasms of mesothelial and soft tissue
C50-C50: Malignant tumor of the mammary gland
C51-C58: Malignant tumors of the female genital organs
C60-C63: Malignant tumors of the male genitalia
C64-C68: Malignant tumors of the urinary organs
C69-C72: Malignant tumors of the eye, brain, and other parts of the central nervous system
C73-C75: Malignant tumors of the thyroid gland and other endocrine glands
C76: Malignant neoplasm with a different and incompletely specified site
C77: Secondary malignant tumor (metastasis) and unspecified malignant tumor of lymph nodes
C78: Secondary malignant tumor (metastasis) of the respiratory and digestive organs
C79: Secondary malignant tumor (metastasis) with other and unspecified sites
C97: Multiple (primary) malignant tumors of different origins
Y43.1: Antineoplastic antimetabolites
Y43.2: Antineoplastic natural remedies
Y43.3: Other chemotherapy
Z29.2: Other Preventive Chemotherapy

Immunosuppressive diseases (including haematological cancer):
B20-B24: Human immunodeficiency virus [HIV] disease
C80: Malignant neoplasm without specified site
C81: Hodgkin's lymphoma
C82: Follicular lymphoma
C83: Non-follicular lymphoma
C84: Mature T/NK cell lymphoma
C85: Other and unspecified types of non-Hodgkin lymphoma
C86: Other Specified Types of T/NK Cell Lymphoma
C88: Malignant immunoproliferative diseases
C90: Myeloma and malignant plasma cell tumors
C91: Lymphocytic leukaemia
C92: Myeloid leukaemia
C93: Monocytic leukaemia
C94: Other leukaemias with specified cell type
C95: Leukaemia with unspecified cell type
C96: Other and unspecified malignant neoplasms of lymphoid, haematopoietic and related tissues
D80-D89: Certain disorders of the immune system
M30-M36: Systemic inflammatory diseases
N16.5: Tubulo-interstitial kidney diseases in graft rejection
T86: Failure and rejection of transplanted organs and tissues

Acute kidney injury:
N17: Acute renal failure

© 2024 Mahalingasivam V et al. *JAMA Network Open*

eAppendix 3. Details of Statistical Model

Below, we have provided details of our statistical model and how the average change in eGFR per year was obtained from model parameters.

Let G denote a particular eGFR measurement, T denote the time at which the eGFR measurement was taken (years since the time of disease infection, with negative values before infection), P denote the binary period indicator of whether the eGFR measurement was taken after disease infection, D denote the type of disease infection (0 for pneumonia, 1 for COVID-19). Finally, let X denote a vector of covariates (potential confounders), as specified in the main text, and let m denote the mean (over individua ls, not eGFR measurements) value of X in the sample.

We used a linear regression model of the form

$$E(G|T,P,D,X) = \beta_0 + \beta_T T + \beta_P P + \beta_D D + \beta_{TP} TP + \beta_{TD} TD + \beta_{PD} PD + \beta_{TPD} TPD + \beta_X X + \beta_{TX} TX.$$

Under this model, the change in eGFR per year, in a particular period P, for a particular disease D and at the average covariate value m, is given by

$$\begin{aligned}\psi(P,D) &= E\{G|T = t+1, P, D, X = m\} - E\{G|T = t, P, D, X = m\}\\ &= \beta_T + \beta_{TP} P + \beta_{TD} D + \beta_{TPD} PD + \beta_{TX} m.\end{aligned}$$

Note that, by including the interaction terms $\beta_{TP} TP + \beta_{TD} TD + \beta_{PD} PD + \beta_{TPD} TPD$, the model allows the change in eGFR per year to vary freely over the four combinations of period and disease.

The difference in eGFR change per year, when contrasting the periods before and after infections, for a particular disease D and a particular covariate level X, is given by

$$\begin{aligned}\phi(D) &= [E\{G|T = t+1, P = 1, D, X\} - E\{G|T = t, P = 1, D, X\}]\\ &\quad - [E\{G|T = t+1, P = 0, D, X\} - E\{G|T = t, P = 0, D, X\}] = \beta_{TP} + \beta_{TPD} D\end{aligned}$$

and the difference in eGFR change per year, when contrasting the two diseases, at a particular period P and a particular covariate level X, is given by

$$\begin{aligned}\phi(P) &= [E\{G|T = t+1, P, D = 1, X\} - E\{G|T = t, P, D = 1, X\}]\\ &\quad - [E\{G|T = t+1, P, D = 0, E(X)\} - E\{G|T = t, P, D = 0, E(X)\}] = \beta_{TD} + \beta_{TPD} P.\end{aligned}$$

To account for the correlation between repeated eGFR measurements from the same individual, we used a clustered sandwich estimator of the variance-covariance matrix V for the estimated model parameters.[1] Since both $\psi(P, D)$, $\phi(D)$ and $\phi(P)$ are linear combinations of the model parameters, the variances of the estimates of these can be obtained from V with standard formulas.

1. Stefanski, L. A., & Boos, D. D. (2002). The calculus of M-estimation. *The American Statistician*, *56*(1), 29-38.

eAppendix 4. Sensitivity Analyses

Sensitivity analysis 1

Description: We restricted our primary outcome analysis to individuals with available creatinine tests both before and after infection (i.e. our secondary dataset).

Justification: We undertook this analysis to compare differences in pre-infection slopes between individuals with creatinine tests before ± after infection (our main analysis), with individuals with available creatinine tests both before and after infection.

Sensitivity analysis 2

Description: We repeated our analysis after excluding individuals who died during the observation period and reported eGFR slopes conditional on surviving.

Justification: We undertook this analysis to explore whether our findings were explained by death (informative censoring).

Sensitivity analysis 3

Description: We repeated our analysis excluding individuals with any history of pneumonia (for both the COVID-19 and pneumonia cohorts).

Justification: We undertook this analysis in case eGFR decline was exacerbated by having previous pneumonia (carryover bias).

© 2024 Mahalingasivam V et al. *JAMA Network Open*

eAppendix 5. Potential Sources of Bias

In the directed acyclic graphs (right): 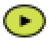 = exposure, 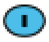 = outcome, 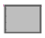 = factor on which selection is conditioned, and 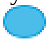 = an alternative cause for the outcome

Death due to pneumonia
Individuals who survived pneumonia may differentially be less likely to experience eGFR decline compared to individuals who survived COVID-19.

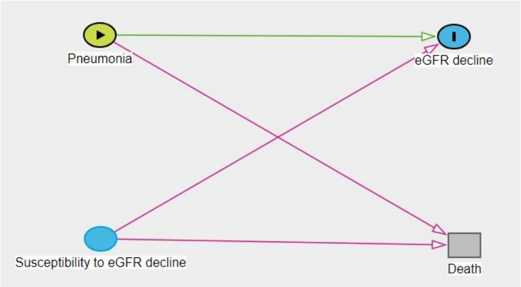

COVID-19 testing
Individuals who are more susceptible to eGFR decline may be more likely to undergo COVID-19 testing due to heightened risk of severe disease.

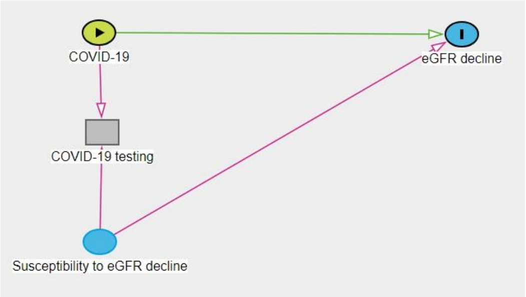

eGFR testing
Individuals who are more likely to have an eGFR decline are more likely to undergo serial eGFR testing.

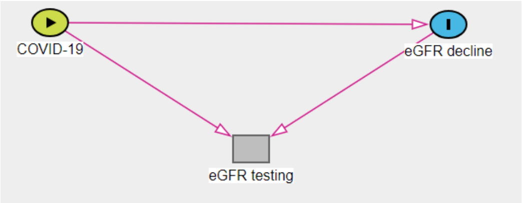

Timing of COVID-19
Individuals with COVID-19 earlier in the pandemic have time available to undergo more eGFR measurements than those with COVID-19 at a later stage, meaning that findings may not be applicable for recent cases.

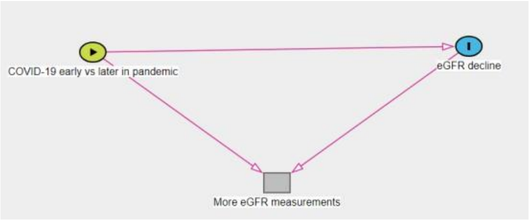

eGFR = estimated glomerular filtration rate

© 2024 Mahalingasivam V et al. *JAMA Network Open*
