# Supplementary material for: Kidney Function Decline After COVID-19 Infection
Source: JAMA Netw Open. 2024 Dec 26;7(12):e2450014. doi: 10.1001/jamanetworkopen.2024.50014 (PMC11672154; doi:10.1001/jamanetworkopen.2024.50014)
Supplement: Supplement 2. — Data Sharing Statement [file jamanetwopen-e2450014-s002.pdf]

Data Sharing Statement

Mahalingasivam. Kidney Function Decline After COVID-19 Infection. *JAMA Netw Open*. Published December 11, 2024. doi:10.1001/jamanetworkopen.2024.50014

Data
Data available: No

Additional Information
Explanation for why data not available: Data cannot be publicly shared due to GDPR Regulations. However, it may be possible to access data through academic collaboration by contacting the principal investigator, Professor Juan-Jesús Carrero, at Karolinska Institute.
